# Suppression of vagal cardiac modulation by blue light in healthy subjects

**DOI:** 10.1186/s40101-016-0110-x

**Published:** 2016-10-05

**Authors:** Emi Yuda, Hiroki Ogasawara, Yutaka Yoshida, Junichiro Hayano

**Affiliations:** Department of Medical Education, Nagoya City University Graduate School of Medical Sciences, Kawasumi 1 Mizuho-cho Mizuho-ku, Nagoya, 467-8601 Japan

**Keywords:** Organic light-emitting diode, Non-image-forming vision, Melanopsin, Intrinsically photosensitive retinal ganglion cell, Blue light, Heart rate variability

## Abstract

**Background:**

In the contemporary life environments, our body is increasingly exposed to various sources of colored light, which may affect our physiological functions as non-image-forming effects. We examined the impacts of colored lights on the autonomic functions by the analysis of heart rate variability (HRV).

**Methods:**

A lighting device consisting of four organic light-emitting diode (OLED) modules (55 × 55 mm^2^) with adjustable red-green-blue color was secured 24 cm above the eyes of subject lying supine in a light-shielded laboratory. Following a 15-min supine rest, electrocardiogram and respiration were measured continuously during 3-min darkness, 6-min colored OLED illumination, and 3-min darkness under paced breathing (15 breath/min). The measurements were repeated at a 45-min interval for red, green, and blue lights with melanopsin-stimulating photon flux density (MSPFD) of 0.00, 0.10, and 0.20 μmol/m^2^/s, respectively, in 12 healthy subjects (23 ± 2 years, two females). Additionally, the effects of blue lights with 0.20, 0.10, and 0.04 μmol/m^2^/s MSPFD were examined in four healthy subjects (25–39 years, two females). HRV was analyzed for low-frequency (LF, 0.04–0.15 Hz) and high-frequency (HF, 0.20–0.30 Hz) power and LF-to-HF ratio (LF/HF).

**Results:**

Compared to darkness before lighting, HF power decreased (*P* < 0.001) and LF/HF increased (*P* = 0.024) during lighting on average of all color lights, whereas HF power showed a greater decrease with blue light than with red and green lights (*P* < 0.05 for both). The decrease in HF power lasted even during darkness after lighting (*P* < 0.001). HF power decreased with blue light with 0.20 μmol/m^2^/s MSPFD (*P* < 0.001) but not with that with 0.10 or 0.04 μmol/m^2^/s (*P* = 0.1 and 0.9, respectively).

**Conclusions:**

Vagal cardiac modulation is suppressed by OLED blue light in healthy subjects most likely through melanopsin-dependent non-image-forming effect.

## Introduction

In the contemporary life environment, our body is increasingly exposed to various artificial lightings with various colors. Light affects many physiological parameters such as melatonin, alertness, body temperature, heart rate, and heart rate variability (HRV) via its non-image-forming visual functions [1–5]. The primary mediator of non-image-forming functions is the melanopsin system whose photoreceptor is intrinsically photosensitive retinal ganglion cells that have a specific sensitivity to blue light around 480 nm [6, 7]. While colored illuminations in our life environments are often selected from esthetic or noticeability aspects, the melanopsin-stimulating property of individual colored illuminations may be important for considering their effects on health and wellbeing.

In this study, we investigated the impacts of colored lights on cardiac autonomic functions by the analysis of HRV with particular interests in whether the melanopsin-stimulating spectral component is the determinant of their effects. For this purpose, we developed a lighting device consisting of organic light-emitting diode (OLED) with adjustable red-green-blue color, which allowed us to generate non-glaring surface illumination with different colors through a single lighting device. Although OLED is expected to be used as a new lighting source for home, occupational, and healthcare environments, there is few study on its physiologic effects.

## Methods

### Subjects

The present study was performed according to the protocol that was approved by the Ethics Review Committee of Nagoya City University Graduate School of Medical Sciences (No. 44-15-0001).

The subjects of this study were recruited with the following inclusion criteria: healthy men or women who (1) were between 20 and 40 years old, (2) had normal color vision, (3) were not taking any medications for >2 weeks, and (4) displayed a normal sinus rhythm on electrocardiogram (ECG) at rest. There were 14 applicants who met the inclusion criteria; 12 of them (mean age ± SD, 23 ± 2 years, two females) participated in study 1, and four of them (age range, 25–39 years, two females) participated in study 2. All subjects gave their written informed consent to participate in this study.

### OLED lighting device

**Fig. 1:**
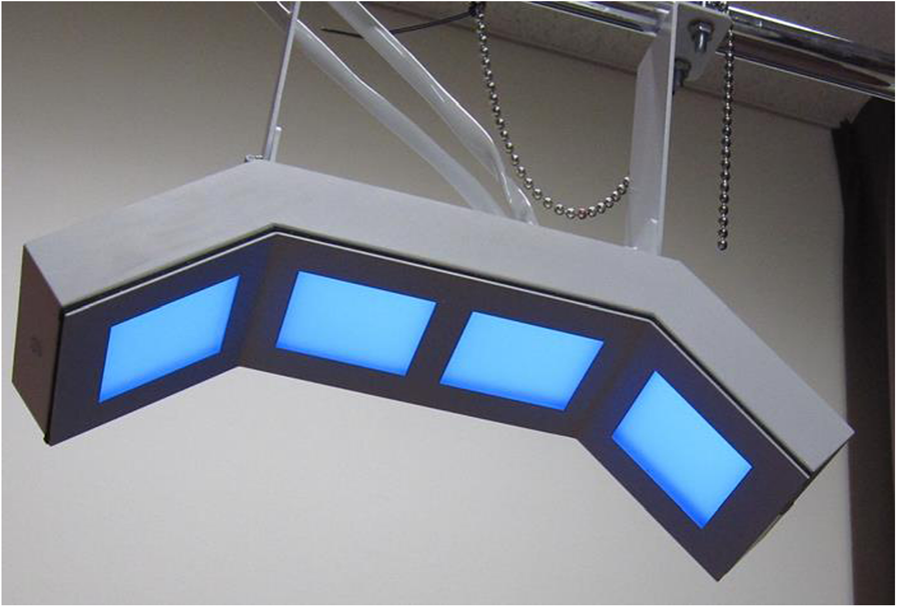
Lighting device with organic light-emitting diode (OLED). The device consists of four OLED panels (55 × 55 mm^2^) with adjustable red-green-blue color and brightness. The panels are linearly aligned and two panels at the both sides incline inward with an angle of 40°

We used an OLED lighting device that was newly developed for the present study (Fig. 1). The device consisted of four OLED panels (VELVE OLED Lighting Module with adjustable red-green-blue color and brightness, 55 × 55 mm^2^, Mitsubishi Chemical Pioneer OLED Lighting Corporation, Tokyo, Japan) that were linearly aligned with two panels at the both sides inclining inward with an angle of 40°. The device was secured 24 cm above the eyes of the subject lying on a bed in the supine position, so that the four OLED panels aligned across the subject’s body axis and the light axes run to the subject’s eyes. We adjusted the lighting device so that it emits three kinds of colored lights (red, green, and blue) and two kinds of dim blue light (5 and 2 lx).

**Fig. 2:**
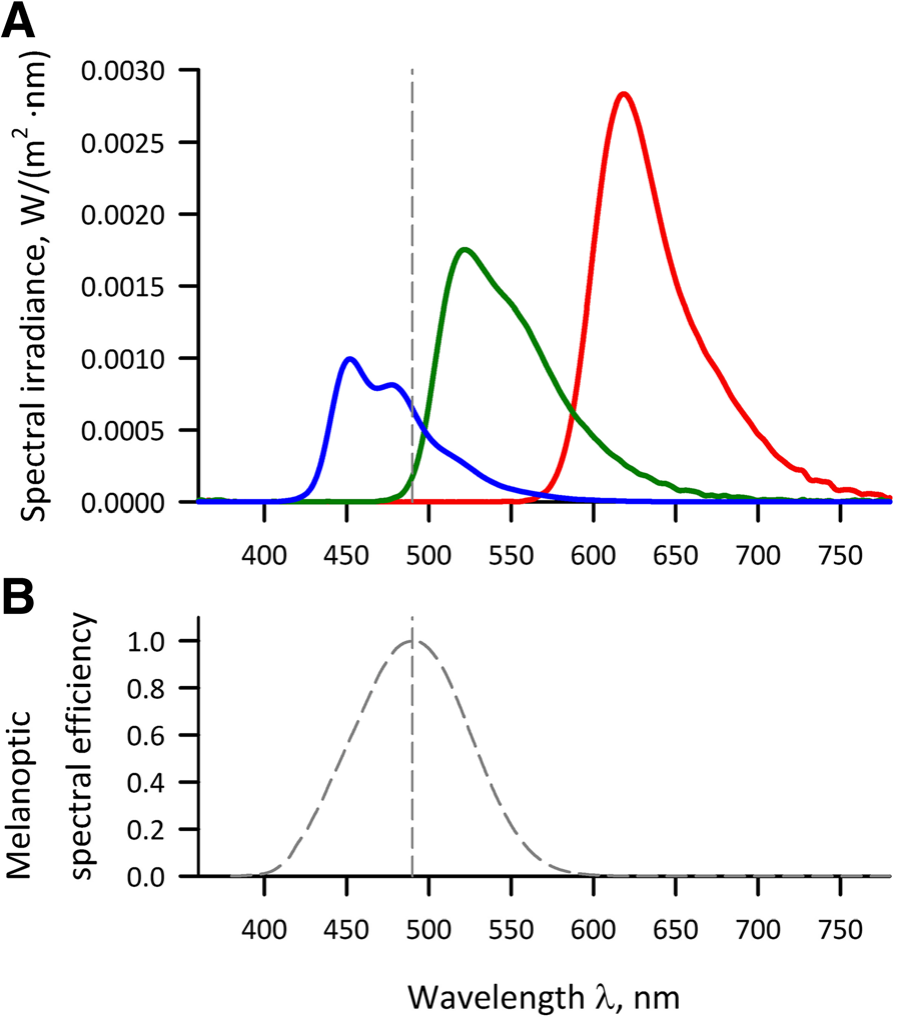
Spectral irradiance distributions of colored OLED lights (**a**) and melanoptic spectral efficiency curve adjusted for human pre-receptoral filtering (**b**). **a** Data were measured at the place of the subject’s eyes, i.e., 24 cm below the surface of OLED lighting module. **b** Generated from data in reference [8]. *Vertical dashed lines* in both panels indicate the position of λ_max_ of the adjusted melanoptic spectral efficiency (490 nm)

Figure 2 shows the spectral irradiance distribution of the three colored lights, and the characteristics of all of these lights are shown in Table 1. The melanopsin-stimulating photon flux densities (MSPFDs) calculated from the *melanoptic* spectral efficiency curve adjusted for the effect of human pre-receptoral filtering [6–8] were 0.00, 0.10, and 0.20 μmol/(m^2^ s) for red, green, and blue lights, respectively, and 0.10 and 0.04 for dim blue lights with 5 and 2 lx, respectively.

**Table 1:** Characteristics of light sources

	Red	Green	Blue^a^	Blue 5	Blue 2
Illuminance, lx	39	71	10	5	2
Irradiance, W/m^2^	0.20	0.14	0.07	0.03	0.01
Chromaticity (*x*, *y*)	(0.63, 0.34)	(0.33, 0.62)	(0.14, 0.16)	(0.14, 0.16)	(0.14, 0.16)
Photon flux density, μmol/(m^2^ s)	1.05	0.64	0.26	0.13	0.05
Melanopsin-stimulating photon flux density, μmol/(m^2^ s)^b^	0.00	0.10	0.20	0.10	0.04

^a^Blue light with 10-lx illuminance was used for both studies 1 and 2

^b^Calculated from melanoptic spectral efficiency adjusted for human pre-receptoral filtering [7, 8]

### Measurements

Lead II ECG and respiration curve with a nose-tip airflow sensor (Dymedix Airflow Sensor, 10-10000-0410, Dymedix Diagnostics Incorporation, Shoreview, MN, USA) were recorded continuously with an 8-channel bioelectric amplifier (Biotop mini, East Medic Corporation, Kanazawa, Japan), digitized at 500 Hz with an analog-to-digital converter (AIO-163202FX-USB, CONTEC Corporation, Osaka, Japan), and stored in a hard disk of a personal computer.

### Study protocols

We performed two studies to examine the effects of difference in the color of light (study 1) and those of difference in the illuminance of blue light (study 2). In both studies, subjects were instructed not to consume food or beverages containing caffeine or alcohol after 21:00 the previous night. The studies were performed between 08:30 and 13:00 in a calm, light-shielded, and air-conditioned (24 ± 2 °C) laboratory more than 2 h after a light meal.

**Fig. 3:**
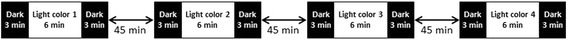
Experimental schedule of dark and illumination conditions. Measurement sessions with three different color lights (red, green, and blue) were performed in all subjects with different orders randomized among subjects at an interval of 45 min

For both studies 1 and 2, data were collected with the experimental schedule of dark and illumination conditions (Fig. 3). In study 1, measurement sessions with three different color lights (red, green, and blue) were performed in 12 subjects with randomized orders among subjects at a 45-min interval. In study 2, sessions with three different illuminance of blue lights (10, 5, and 2 lx) were performed in four subjects with randomized orders at a 45-min interval. The 10-lx blue light used in study 2 was the same as blue light used in study 1.

At each session, the subjects lied on the bed in the supine position, so that their eyes were right below the OLED device, and wore a headphone for the purpose of paced breathing. We used paced breathing method to prevent the changes in breathing frequency from confounding the assessment of vagal cardiac modulation by HRV [9, 10]. We developed a computer program that generates an audio signal consisting of high (960 Hz)- and low (770 Hz)-pitched sounds, which appeared alternatively for 2 s each (at an interval of 4 s), resulting paced breathing at 15 cycle/min (0.25 Hz). The subjects were instructed to breathe in when hearing high-pitched sound and to breathe out during low-pitched sound. Before the experiment, all the subjects practiced the paced breathing until they become able to breathe comfortably in synchrony with the audio signal coming through the headphone.

For both studies, measurement of each session was started after a 15-min supine rest. The subjects were instructed to keep their eyes open, to gaze at the OLED panels, and to continue the paced breathing throughout each measurement session.

### Data analyses

Data were analyzed off-line on a personal computer. The temporal positions of all QRS waves in digitized ECG data were detected with a fast-peak detection algorithm. After all errors in the detection of QRS waves were edited, time series of the R-R interval were obtained. The R-R interval time series thus obtained were analyzed separately for the periods of three different conditions (3-min darkness before lighting, 6-min lighting, and 3-min darkness after lighting) in individual measurement sessions with four different lighting colors.

For each data segment, frequency domain analyses of the HRV were performed with fast Fourier transformation (FFT) with the original software in our laboratory [9]. Briefly, R-R interval time series were interpolated with a step function only using interval data consisting of consecutive QRS waves in sinus rhythm, resampled at 256 and 512 equidistant time points for 3-min and 6-min data segments, respectively, filtered with a Hanning window, and converted into frequency domain by FFT. After correcting for the losses of variance resulting from the sampling and filtering processes, the absolute power of the low-frequency (LF, 0.04–0.15 Hz) and the high-frequency (HF, 0.15–0.40 Hz) components were computed. The powers of LF and HF components were transformed into natural logarithmic values. We used the HF power as an index of vagal cardiac modulation for cardiopulmonary resting [9, 11, 12].

The respiration data were also analyzed by FFT, and breathing frequency was estimated from the position of the dominant spectral peak. The synchronicity of respiration was evaluated from the breathing frequency, and data were excluded from this analysis if it deviated from the range between 0.23 and 0.27 Hz.

### Statistical analysis

Statistical analyses system version 9.4 (SAS institute, Cary, NC, USA) was used for the statistical analysis. Our primary interest in study 1 was to clarify whether the autonomic neural activities during OLED lighting differ with the color of lighting and the secondary interest was to examine the effects of colored lighting on the autonomic activity after exposure to lighting. For these purposes, two-way repeated measures ANOVAs of autonomic indices were separately performed on the difference between before and during lighting and on the difference between before and after lighting. For study 2, to evaluate the effects of blue light with different illuminance, we used paired *t* test for each illuminance. *P* < 0.05 was considered to be statistically significant, and Bonferroni method was used to keep type 1 error level of <0.05 in multiple comparisons.

## Results

**Fig. 4:**
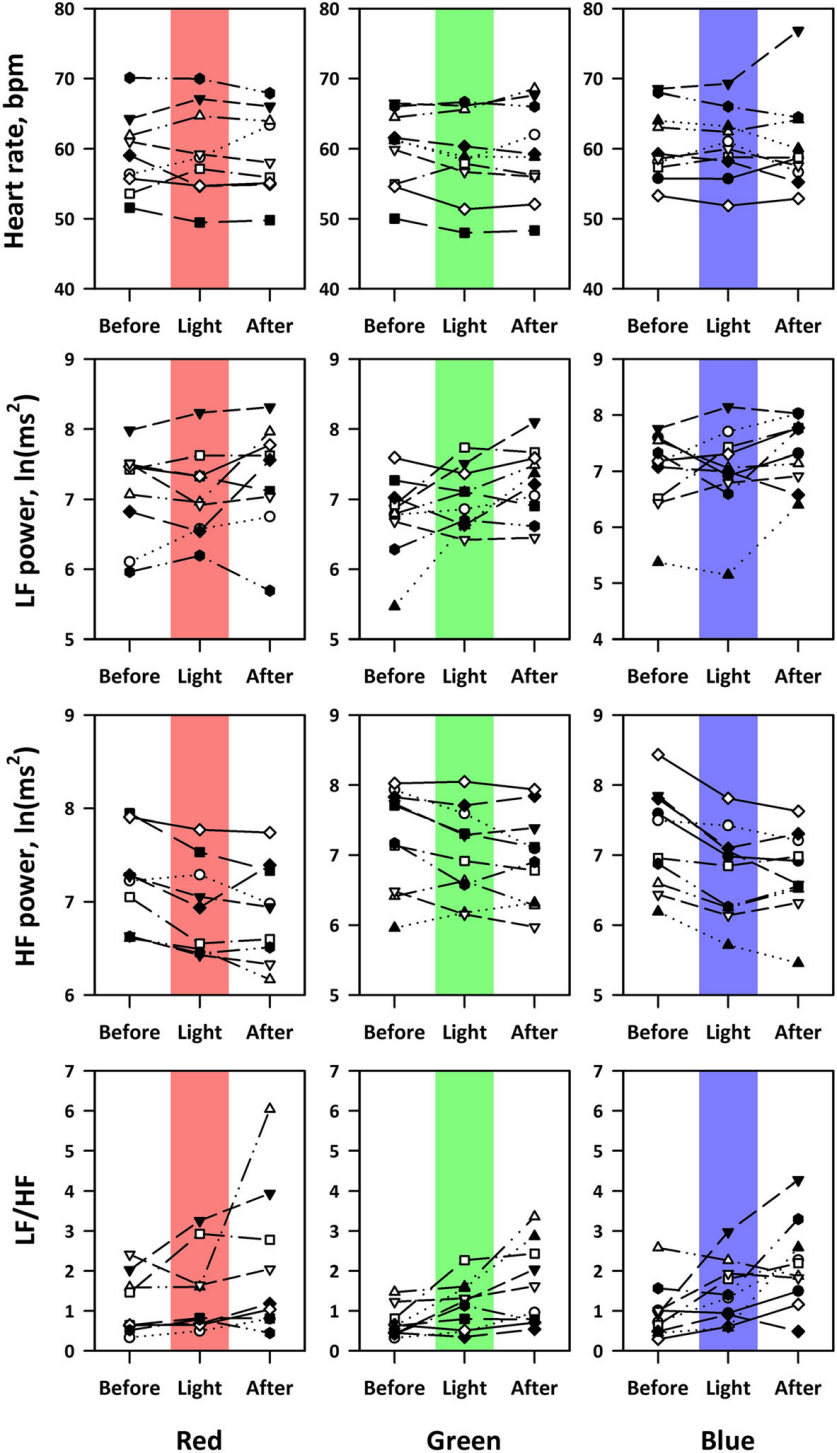
Heart rate and heart rate variability (HRV) indices in individual subjects before, during, and after exposures to red, green, and blue OLED lights. *HF* high-frequency component, *LF* low-frequency component, *LF/HF* LF-to-HF ratio in power

In study 1, we used data in 10 subjects (mean age ± SD, 24 ± 1 years) out of 12, because two subjects (one male and one female) were excluded due to the loss of respiratory synchronicity. The heart rate and HRV indices in individual subjects before, during, and after exposures to red, green, and blue OLED lighting are shown in Fig. 4.

**Fig. 5:**
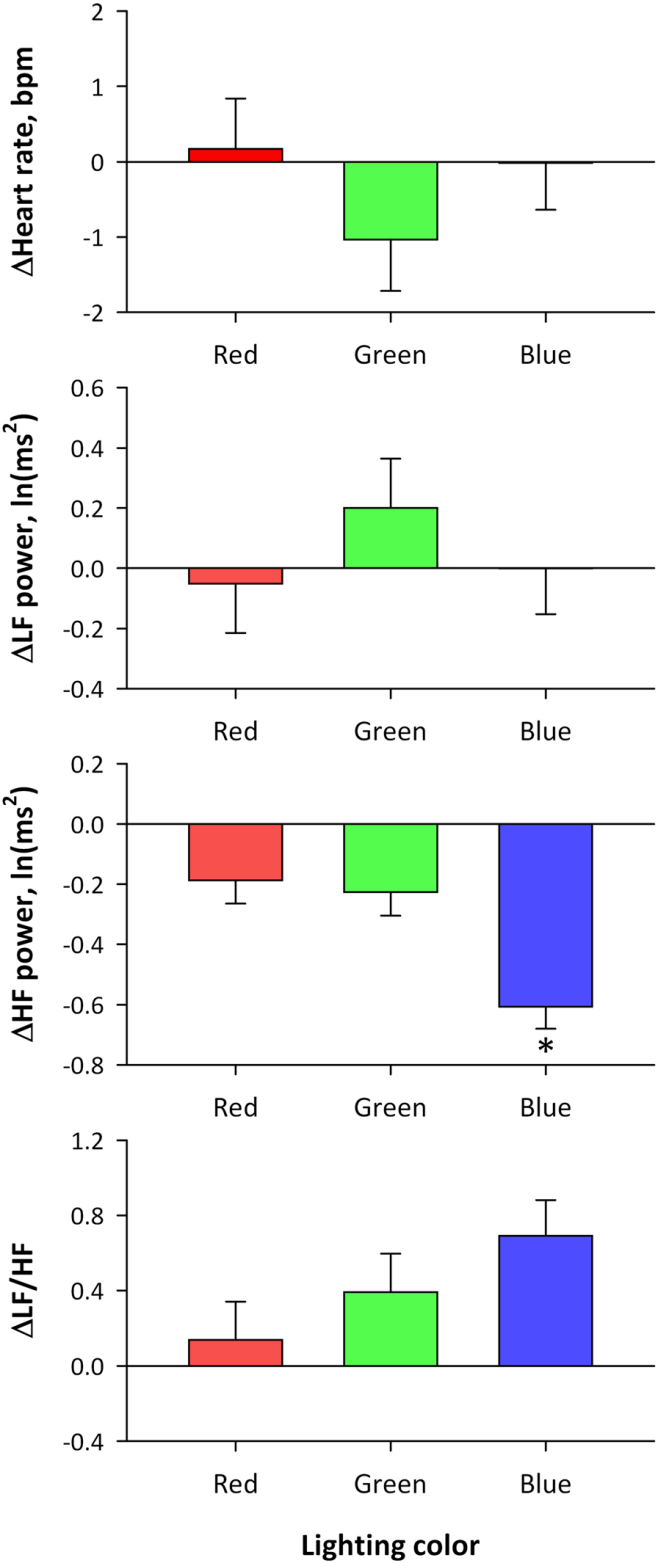
Average changes in heart rate and HRV indices from before to during OLED lighting. *Error bars* represent standard error of mean. *Significantly different from values for red and green with multiple comparisons (*P* < 0.05)

Repeated measures ANOVA on the difference between before and during lighting revealed that there was no significant main effect of color or time (before and during lighting) or their interaction on heart rate or LF power, whereas significant main effects of color and time and their interaction on HF power and significant main effect of time on LF-to-HF ratio (LF/HF) were observed (Table 2). As shown in Fig. 5, HF power decreased and LF/HF increased during lighting. Multiple comparisons of HF power that showed significant time × color interaction indicated that the decrease in HF power with blue light was greater than those with red and green lights (*P* < 0.05 for both) with no significant difference between those of red and green lights (Table 3).

**Table 2:** Repeated measures ANOVA on differences in heart rate and HRV indices between before and during lighting

	Main effect				Interaction	
	Color (DF = 2)		Time (DF = 1)^a^		Time × color (DF = 2)	
	*F* value	*P*	*F* value	*P*	*F* value	*P*
Heart rate	0.07	0.93	2.83	0.12	1.30	0.30
LF power	0.52	0.60	0.01	0.93	0.58	0.57
HF power	4.62	0.030	42.04	<0.001	6.52	0.010
LF/HF	1.70	0.22	6.52	0.024	0.92	0.42

*DF* degree of freedom, *HF* high-frequency component, *LF* low-frequency component, *LF/HF* LF-to-HF ratio in power

^a^Effect of time between before and during lighting

**Table 3:** Multiple comparisons of the changes in HF power (during-before) between colors of lighting

	Red	Green	Blue
Red	–	0.75	0.007^a^
Green	0.75	–	0.010^a^
Blue	0.007^a^	0.010^a^	–

Values are *P* value for the significance of difference between two colors

^a^Significant after Bonferroni adjustment (*P* < 0.05)

**Fig. 6:**
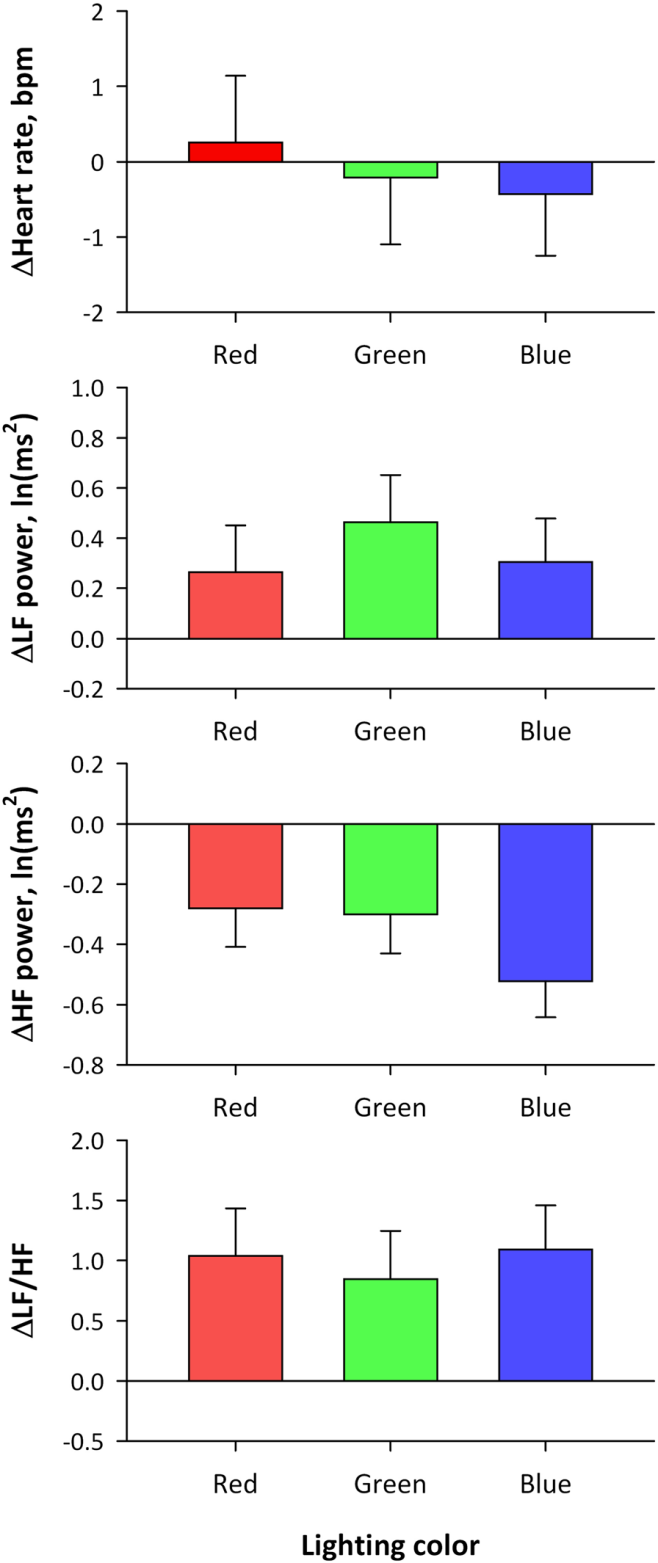
Average changes in heart rate and HRV indices from before to after OLED lighting. *Error bars* represent standard error of mean

Repeated measures ANOVA on the difference between before and after lighting revealed significant main effects of time on LF and HF power and LF/HF (Table 4). Although significant main effect of color was also revealed on HF power, no significant interaction between time and color was detected. As shown in Fig. 6, LF power and LF/HF were increased and HF power was decreased after lighting.

**Table 4:** Repeated measures ANOVA on differences in heart rate and HRV indices between before and after lighting

	Main effect				Interaction	
	Color (DF = 2)		Time (DF = 1)^a^		Time × Color (DF = 2)	
	*F* value	*P*	*F* value	*P*	*F* value	*P*
Heart rate	0.07	0.93	0.27	0.62	0.06	0.94
LF power	0.36	0.70	6.67	0.022	0.29	0.75
HF power	4.63	0.030	22.38	<0.001	1.98	0.18
LF/HF	1.70	0.22	12.71	0.004	0.17	0.84

Abbreviations are explained in the footnote found in Table 2

^a^Effect of time between before and after lighting

**Fig. 7:**
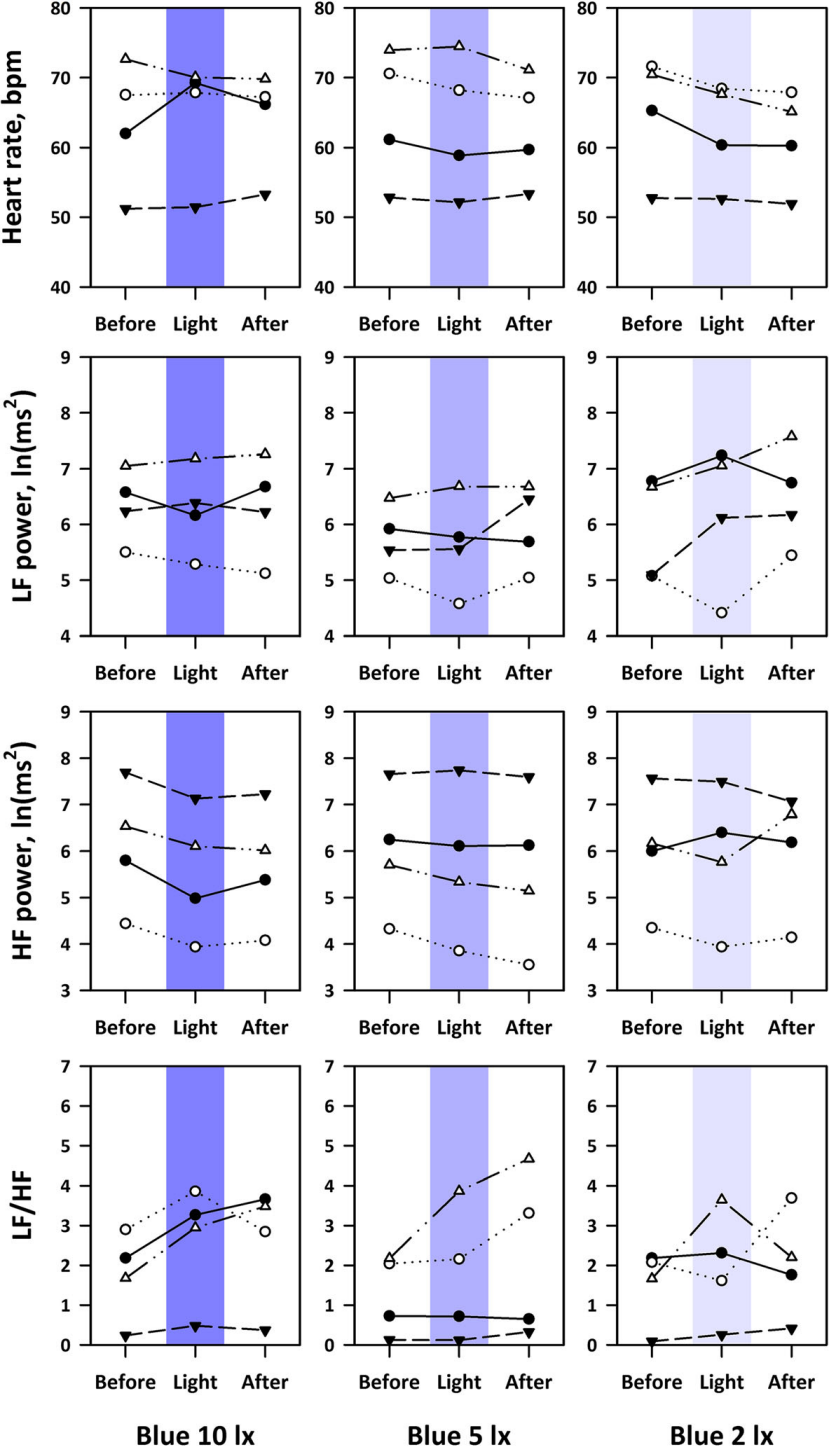
Heart rate and HRV indices in individual subjects before, during, and after exposures to blue OLED lights with 10, 5, and 2 lx

In study 2, respiratory synchronicity was maintained in all subjects. Figure 7 shows the heart rate and HRV indices in individual subjects before, during, and after exposures to blue lights with 10, 5, and 2 lx (MSPFD, 0.20, 0.10, and 0.04 μmol/m^2^/s, respectively). Blue lights with 5 and 2 lx caused no significant changes in heart rate or any of HRV indices either during or after lighting, while blue light with 10 lx increased HR during darkness after lighting (*P* = 0.03), decreased HF power both during lighting (*P* = 0.006) and darkness after lighting (*P* = 0.001), and increased LF/HF during lighting (*P* = 0.02).

## Discussion

To investigate the acute physiological effects of colored OLED lights in healthy young subjects, we looked into the changes in HRV indices during and after exposure to lights with different color and illuminance. We found that blue light caused a greater decrease in HF power than red and green colors. While the blue light had a lower illuminance (10 lx) than red and green (39 and 71 lx, respectively), it had the highest MSPFD (0.20, 0.10, and 0.00 μmol/(m^2^ s) for blue, green, and red, respectively). Furthermore, the decrease in HF power was observed for 10-lx blue light but not for 5- or 2-lx blue lights, indicating that the decrease in HF power with blue light is not attributable to its lower intensity. The decrease in HF power was also observed even during darkness after the termination of lighting. These findings support the hypothesis that blue light suppresses vagal cardiac modulation through melanopsin-dependent non-image-forming effect. Also, the sustained response of vagal suppression during darkness after lighting seems also consistent with the property of the melanopsin-dependent non-image-forming effect that is known to have sustained response after light exposure [13, 14].

Earlier studies have reported mixed results for the effects of colored fluorescent lights on HRV [15, 16]. For example, Schäfer et al. [15] have analyzed the changes in HRV with 10-min exposure to red, green, and blue fluorescent light of 700 lx preceded and followed by 15-min darkness in 12 healthy young subjects. Although they failed to detect significant change in absolute HF power during exposure to any color of lights, their results indicated a significant decrease in absolute HF power during darkness after exposure to blue light, while there were no significant changes with exposures to red or green lights. Their results seem partially consistent with ours, suggesting a specific sensitivity of the HF component to blue light. Choi et al. [16] have also analyzed HRV before and after 5-min exposure to blue, red, and white fluorescent light in 92 healthy adults in the seating position with spontaneous breathing. They observed a decrease in absolute HF power after exposure to red light, while there was no significant change with exposure to blue or white light. Although their results seem inconsistent with ours, the difference in body positions during HRV measurement between the studies may be critical because the HF power is strongly suppressed by gravitation stress [17].

In the present study, we used paced breathing for the autonomic assessment by HRV. Although HF component of HRV is widely used as an index of vagal cardiac modulation [11, 18], the power of this component is also affected by respiration frequency; HF power decreases with increasing respiration frequency independently of cardiac vagal modulation [10, 19]. Furthermore, while the HF component is thought to be generated centrally by the mechanism of cardiorespiratory coordination, earlier studies have suggested that this central coordination may be affected by light depending on its color [20]. Because the changes in HF power in the present study were observed under paced breathing, they may be interpreted to reflect the direct effects of colored light on central vagal function. In contrast, the changes in HF power under spontaneous breathing could be indirectly mediated by the changes in respiratory frequency at least partly.

In study 1, we compared the autonomic effects among red, green, and blue OLED lights with different intensity (illuminance, irradiance, and photon flux density); the blue light was the lowest in these parameters (Table 1). Thus, we were unable to determine whether the observed differences between blue light and other colored lights were caused by the color of lights or their intensity. The method for standardizing the light intensity for comparing the effects of colors of light has not been established [8]. Also, possible changes in pupillary size with light could affect the amount of light reaching the retina, even if the intensity of lights was standardized in some way. Thus, we instead investigated the effects of intensity of blue light to examine whether the autonomic effect of blue light is caused by its low intensity or not (study 2). We observed that the suppression of HF power occurred only with 10-lx blue light but not with 5-lx or 2-lx blue lights. Additionally, the estimated MSPFD of 5-lx blue light was the same as that of 71-lx green light. These indicate that the greater suppression of HF power with the 10-lx blue light than with the red and green lights is not attributable to the lower intensity of blue light and the pattern of responses suggests that the suppression of HF power by blue light is most likely explained by its higher MSPFD.

While HF power decreased with 10-lx blue OLED light, heart rate showed no significant change in study 1 and only a modest increase in study 2. Although HF power has been often interpreted simply as an index of cardiac vagal tone to control heart rate, HRV in HF band, particularly under paced breathing at >0.15 Hz, is a quantitative reflection of respiratory sinus arrhythmia (RSA). We have previously reported that RSA is a cardiopulmonary resting function for saving cardiac and respiratory energy by suppressing unnecessary heartbeats during expiration and ineffective ventilation during waning phases of perfusion [12, 21]. Although the magnitude of RSA and heart rate usually change reciprocally to each other with physical and mental stress and relaxation, they are thought to be regulated separately by the vagal outflows from the nucleus ambiguous and the dorsal motor nucleus of vagus, respectively; the former generates phasic changes with respiration, while the latter causes tonic pattern [22]. These suggest that blue OLED light may suppress the vagal mechanisms generating RSA without affecting substantially the vagal mechanisms controlling heart rate. One may speculate that blue OLED light might have an effect that shifts the state of our body to arousal from resting.

We used a newly developed OLED lighting device for this study. Because we did not compare the OLED with conventional lighting sources including fluorescent lamps and light-emitting diode (LED), we were unable to determine whether the results we observed are specific to OLED or not. As shown in Fig. 2, however, the spectral irradiance of OLED blue light has a broader spectrum at the region of the melanoptic spectral efficiency curve [6–8] compared with those reported for blue LED [23] and for fluorescent lamps [24]. OLED is gathering attentions as non-glaring comfortable surface illumination and is expected to be used as a new lighting source at home, workplace, and healthcare environments. Our findings of the autonomic effects seem important characteristics of colored OLED illuminations, which should be considered on their utilization.

### Limitations

This study has several limitations. First, we examined the effects of lighting only for 6 min. Although we observed a significant reduction in HF power both during and after the exposure of blue light, it is not clear whether the response had been saturated during 6 min or might have progressed more by longer exposure. The same is the case for recovery of the response. We were unable to determine the time course of the recovery. If the response last long, the 45-min interval between the measurements with different color lights might have been insufficient for washing out the effects of the previous exposure. Second, we did not standardize the intensity of illumination among different colors. Thus, we cannot exclude the effects of the intensity on our results. However, as discussed above, the suppression of HF power by blue light was not explained by its low intensity in study 2. Third, because we did not measure melatonin secretion in this study, we were unable to determine whether the colored lights affect the entrainment to environmental light-dark cycles or not. Although our findings seem consistent with the hypothesis that blue light suppresses vagal cardiac modulation through melanopsin-dependent non-image-forming effect, it does not exceed the range of speculation. Finally, this is a preliminary study with a small sample size. To determine the effects of gender and aging on the results, future studies are necessary.

## Conclusions

We examined the impact of OLED colored lights on HRV in healthy young subjects under paced breathing. Our observations indicate that vagal cardiac modulation is suppressed by OLED illumination as a direct response not mediated by respiratory frequency. Also, this response may be more sensitive to blue light than to red and green OLED lights and may last even after the exposure. This study suggests that on the use of colored OLED lighting in a variety of environments, we need to consider the difference in physiological effects with the color of illuminations.

## Abbreviations

ECG: Electrocardiogram
FFT: Fast Fourier transformation
HF: High frequency
HRV: Heart rate variability
LF: Low frequency
LF/HF: LF-to-HF ratio
MSPFD: Melanopsin-stimulating photon flux density
OLED: Organic light-emitting diode
RSA: Respiratory sinus arrhythmia
